# Case of Survival of Twelve Days' Abstinence from Food, Occurring in a Person in the State of Health

**Published:** 1820-02

**Authors:** Thomas T. Griffith

**Affiliations:** Member of the Royal College of Surgeons.


FOR THE LONDON MEDICAL AND PHYSICAL JOURNAL.

### *Case of Survival of Twelve Days' Abstinence from Food, occurring in a Person in the state of Health.* By Thomas T. Griffith, Member of the Royal College of Surgeons.

THE leading particulars of the following extraordinary case, having happened in part under my own immediate observation, and in part under such circumstances as leave but little room to doubt their having occurred, I am induced to request a place for the insertion of them in your Journal.

On the morning of the 27th of last September, an immense body of water, which had accumulated in one coal-pit, suddenly burst into another, where nineteen men were at work; sixteen of them were enabled to extricate themselves from the perilous situation which they were thus unexpectedly placed in, at a distance from the surface of one hundred and twenty yards. But, such was the quantity of water, and the changes it rapidly produced in the pit, that the remaining three were cut off from all means of escape. In the hope of being able to rescue these unfortunate people, every measure that could possibly expedite the discovery of them was unceasingly pursued. On the ninth day the bodies of two, who had been drowned, were discovered, and on the twelfth day some vestiges of the third man were traced; in a short time his voice was heard; and soon afterwards he presented himself to the astonished workmen.

It appeared from his subsequent statement, that, when the water was rushing in an overwhelming stream towards the spot where he stood, he had only sufficient time to allow his gaining a situation above the highest level to which the water reached. In this place, which was a dark, low, close passage, communicating with two galleries, he remained for *twelve days*, without any other sustenance than a little water, which, trickling down a rock distant about twelve paces from the place where he lay, he collected in a hollow formed by his hand; to this he occasionally crept on his hands and knees, and at other times he relieved himself by merely changing his posture. He computed the period of his confinement to have been about *nine* days. For the first third part of this time he felt a considerable desire for food, which afterwards entirely ceased; and he only experienced an occasional painful sensation in the region of his stomach, which was always removed by drinking a little water. The greatest part of the time was spent in sleep, accompanied by dreams. Some of the functions of the body appear to have been nearly suspended, and others to have been very slowly performed. The bowels were relieved once only; there was no sensible perspiration; the breathing was slow; but the urine was voided in small quantities about twenty times. His mind suffered but little depression, except upon one point; in general, it was at ease, and he felt confident that at the end of a computed time the water would be removed, and a communication with himself be opened: but, when he reflected upon the destitute and unhappy state of his wife and children, the powers of reason yielded to a pleasing hallucination: he thought that celestial sounds were near him; every unpleasant feeling ceased, and happier and more animating views employed his thoughts.

At the moment he was found, and for some time afterwards, he declined taking any thing more than a little dry biscuit; he was therefore merely removed to a warmer part of the pit, where I first saw him. He was in full possession of his mental faculties, his spirits were buoyant, and his feelings alive to the hearty congratulations of his companions, and to the anticipated pleasure of returning to his family. His face and figure were much emaciated;* there was an expression of wildness and famine in his countenance; his cheeks were flushed; his tongue was slightly furred and white; his pulse was regular, about 112, but very weak and small; and his extremities were cold. He complained of some degree of thirst, but of no pain: he had no wish to take food.

Fearful of calling into sudden or violent action the accumulated irritability of the system, liquid food, of a mild nutritive kind, was administered to him in small quantities, and at stated intervals: although he took it with indifference, his stomach retained it. The circumstance of his deliverance, and his anxiety to see his family, excited his mind so powerfully, as to give an unexpected degree of tone and energy to his whole frame; of this I thought it right to avail ourselves, and to remove him from his present situation to some house, where we could better guard against the effects of that debility which might be expected speedily to result from his present state. He was brought up from the pit in a tub, so large as to admit of his lying down in it, lest a continuance in the erect posture might have induced syncope, under circumstances very unfavourable to his recovery. In this way he experienced very little inconvenience from his removal. During that evening, and the following night, he was ordered to take the yolk of egg, mixed with warm water and sugar, weak broths, gruel, milk, &c. in larger quantities and with longer intervals; and, once in three hours, part of a slightly-cordial mixture, containing a few drops of laudanum in each dose. The nurse was directed to wake him occasionally, if much disposed to sleep.

* On the fourth day from that on which he was found, he weighed twenty-eight pounds less than he had done some weeks previously. He is a muscular bony man, of the middle size.

*Sunday*, (the following morning.)—He has passed a tolerably comfortable night, occasionally slumbering. He has taken some quantity of food without any sickness. He now feels much exhausted; his pulse is languid, and reduced from 112 to 60 strokes in a minute. He has no desire for food: faintness comes on in the erect posture. He has voided urine. A more nourishing and stimulating diet was ordered. In the evening he was better, but suffered some degree of uneasiness from flatulency and heat. As his bowels had not been relieved for some days, the following mixture was prescribed: ℞. Pulv: Rhei ʒij. Potassæ Tartr. ʒj. Tinct. Sennæ ʒiij. Aquæ Puræ Infus. Rhei à ℥iss. M. Capiat cohlearia ij. Majora 3ta. quâque horâ, donec alvus respondeat. The operation of the medicine produced four evacuations of hardened black fæces, and afforded the patient much relief.

*Monday.*—He is better; has passed an easy night with some sleep. His pulse is 84, and fuller. His appetite is returning. He complains of an unpleasant degree of heat in his feet. The same kind of diet is to be continued.

*Tuesday.*—This morning I was requested to visit him as soon as possible, as he was much worse. I was informed, that, finding himself better on the preceding evening, and his appetite greater, he had taken solid food, and ale in small quantities: in consequence of which he had had a very restless night; his skin had been hot and dry; his bowels distended and painful; his stomach oppressed; he had been thirsty, and at times delirious. Nor did these symptoms disappear, until the stomach had been relieved of its contents, and the bowels had been acted upon by an aperient. Two or three days of restlessness, heat, and languor, with diminished appetite, elapsed, before he recovered that point of convalescence which he had thus lost by his own temerity and imprudence. Such a degree of reaction took place in his feet and toes, as seemed for a short time to threaten the vitality of those parts.

During the ten or twelve ensuing days, a variety of symptoms, chiefly arising from the different states of the alimentary canal, appeared. For a day or two the pulse intermitted, but was restored to its regular action by emptying the bowels. Nothing material, however, prevented his ultimate recovery; and his general health is now (Dec. 16th,) perfectly re-established. His last complaint was of a severe rheumatic pain in the shoulder, on which he had lain during the greater part of the period of his confinement in the pit, and from which he still continues to suffer at times.

*Remarks.*—It may be asked, "were there any peculiar or local circumstances which could have aided the powers of the system in supporting life, for so long a period, under a deprivation of food, so entire?" I believe that, providentially, there were. The man's age 27; his bodily strength, his habits, and that self-possession and strength of mind which led him to an almost correct calculation of the time required for the removal of the water and other obstacles, and enabled him to wait patiently the expiration of that time, in a confident hope of being ultimately rescued, were advantages greatly in his favour. But there existed also a local cause,—the peculiar kind of air which he breathed; and which, I am disposed to think, contributed materially to the preservation of life. From repeated examinations of the air in the pit, from my own feelings and observations whilst below, and from the man's description, it was ascertained that, with the water, a quantity of carbonic acid gas had diffused itself through the lower parts of the pit. The situation which the man occupied was an elevated one, and would therefore raise him above the more concentrated stratum of carbonic acid gas; but he was still aware that the surrounding air was heavy and oppressive: he said, "that for the greater part of the time he breathed the *damp* air, but that it became better as the water lowered." To enter again upon the particulars of the case, or to reason upon the known properties of carbonic acid gas, and its effects under different modifications, would extend these observations beyond their proper limits. From the positive evidence of the man having breathed an atmosphere containing a greater or less proportion of carbonic acid gas, for twelve days, and from his having survived, may we not infer, that this gas, by producing a sedative effect on the system, by moderating irritability, and restraining action, prevented the active exercise, and consequent exhaustion of the vital powers, and thus proved conducive to the preservation of life?

*Wrexham, December,* 1819.

